# Leaf Length Predicts Twig Xylem Vessel Diameter Across Angiosperms

**DOI:** 10.1111/pce.70287

**Published:** 2025-11-24

**Authors:** Patricia Rivera, Tommaso Anfodillo, Mark E. Olson

**Affiliations:** ^1^ Instituto de Biología Universidad Nacional Autónoma de México Coyoacán Ciudad de México Mexico; ^2^ Present address: Centro de Investigación Científica de Yucatán A.C. Calle 43 No. 130 x 32 y 34 Chuburná de Hidalgo. CP 97205 Mérida Yucatán Mexico; ^3^ Department Territorio e Sistemi AgroForestali University of Padova Padova Italy

**Keywords:** adaptation, conduit widening, vessels, xylem hydraulics

## Abstract

As plants grow taller, increasing conductive pathlength imposes hydraulic resistance, challenging the maintenance of water transport to leaves. While tip‐to‐base conduit widening along the stem helps mitigate this resistance, theoretical models and empirical data suggest that stem widening alone is insufficient to fully compensate. Here, we explore whether leaf length could contribute to maintaining hydraulic conductance by influencing vessel diameters in the stem. Across a diverse set of angiosperm species, we found that leaf length strongly predicts vessel diameter at the petiole base, and that petiole vessel diameter, in turn, scales positively with vessel diameter at the twig tip. These relationships imply that longer leaves are associated with wider conduits in the stem, potentially boosting stem‐wide permeability. Simple fluid dynamic models show that the steep rate of conduit widening in angiosperm leaves plausibly buffers the resistance costs of increased leaf length. Because vessel diameter scales with the fourth power of conductance, modest increases in leaf length, and thus stem conduits, could lower the resistance not buffered by conduit widening in the stem. Leaf length during height growth may serve as a key mechanism in maintaining hydraulic supply, complementing conduit widening in the stem.

## Summary statement

Longer leaves in woody angiosperms have wider petiole vessels, which in turn predict wider twig tip vessels. This linkage may boost stem‐wide conductance, complementing tip‐to‐base widening in maintaining water supply as plants grow taller.

## 1 Introduction

The diameter of conduits in the xylem of stems is an important variable in maintaining conductance to the leaves as plants grow in height. In the conductive system of a plant, the narrowest conduits tend to be found at the terminal ends of the conductive stream, the “capillaries” in the leaves where water diffuses out of the long‐distance xylem transport stream into the leaf mesophyll. Narrow conduit diameters at the terminal end of the conductive stream maximize the surface area across which a given fluid volume can diffuse (West et al. 1999a). As the length of a tube increases, the resistance to fluid flow also increases, leading to a drop in conductance. This is a fundamental principle in fluid mechanics and applies to both Newtonian and non‐Newtonian fluids (Vogel 1994; Lambride et al. 2023). So if diameters remained constant from these narrow termini down through the petiole and into the stem, then as plants grow taller and the conductive pathway becomes longer, conductance would steadily drop. In this way, conductive pathlength represents a significant selective pressure shaping the whole plant conductive system. Conductance scales as the fourth power of conduit diameter, so one adaptive response is that in terrestrial plants, conduits widen along the stem, from the twig tip (the apical meristem) down through the branches and into the trunk (Koçillari et al. 2021). Given this fourth‐power relation, even relatively subtle widening is sufficient to offset to a large degree the resistance that accumulates linearly with increases in conductive path (West et al. 1999b; Becker et al. 2000; Petit and Anfodillo 2009).

**Figure 1:**
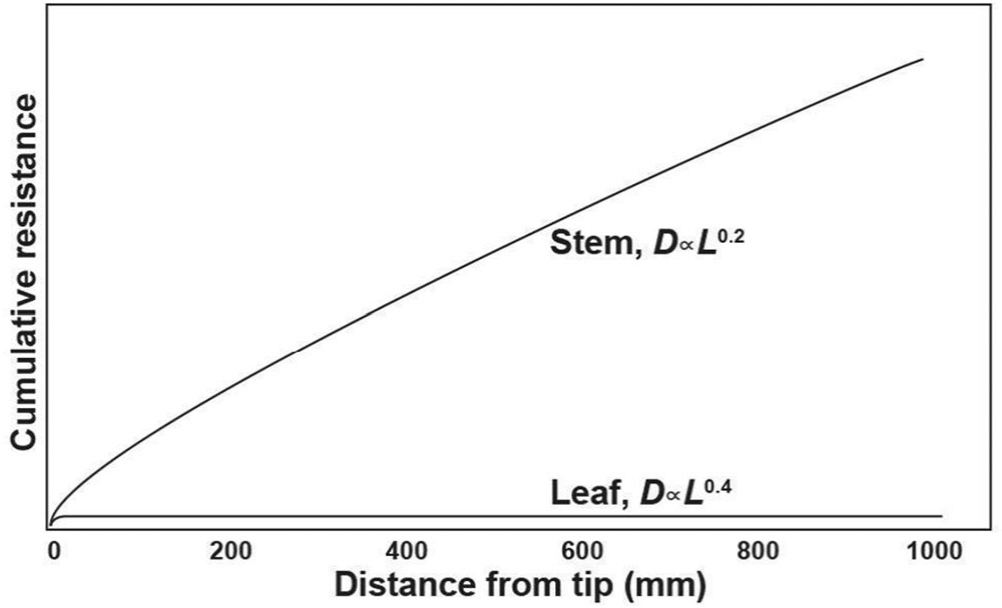
Cumulative resistance in leaves is nearly independent of path length, whereas in stems it rises more steeply with distance from the tip. The X‐axis shows distance from the tip, either the distalmost leaf veins or the twig tip, in millimeters. The Y‐axis shows arbitrary resistance units derived from a simple model based on stacked cylindrical conduits and Poiseuille's law. The graph can be interpreted as a horizontal path from tip (left) to base (right). It illustrates how conduit widening in stems, *D*_*twig*_∝*L* ^0.2^, substantially reduces the resistance that would otherwise accumulate if diameter remained constant. However, the widening rate in angiosperm leaves is much steeper, *D*_*pe*t_∝*L* ^0.4^, making resistance in leaves nearly independent of leaf length.

However, empirical scaling exponents suggest that stem conduit widening alone does not fully compensate for the increase in resistance associated with height growth (Petit and Anfodillo 2009). Across terrestrial plants, tip‐to‐base conduit widening approximates a power law, with conduit diameter (*D*) widening with length of the conductive path (*L*), measured as the distance from the twig tip, as *D∝L* ^*b*^ *. b* in stems varies from 0.1 to 0.3 (Koçillari et al. 2021). This widening greatly ameliorates resistance, but widening in stems is not sufficient on its own to buffer all of the resistance associated with height growth (Figure 1). Therefore, other compensatory mechanisms in addition to widening must exist if plants are to maintain conductance to their leaves with height growth; we have called these beyond‐widening compensations “ultra‐widening permeability” mechanisms (Anfodillo and Olson 2024).

A major potential source of increase in ultra‐widening permeability with height growth is widening of terminal twig conduits. Given two individuals of identical height, both with the same rate of tip‐to‐base conduit widening, for example *D∝L* ^*0.2*^, if one individual has wider terminal conduits, then that individual will also have wider conduits along the entire conductive path. Given Poiseuille's law assumptions, one individual with twig tip conduits that are 10 µm in diameter, and the other has conduits that are 20 µm in diameter, all else being equal, the one with wider conduits will have a conductance 16 times higher, with just a 10 µm difference in terminal conduit diameter.

Therefore, it is possible that in concert with tip‐to‐base conduit widening, small increases in terminal twig conduit diameter could maintain conductance constant as a tree grows taller. Assuming a scaling relation of conduit diameter with path length as *D∝L* ^0.2^, and flow assuming Poiseuille's law, an individual 10 m tall with terminal twig conduits 18.2 µm in diameter would have the same cumulative hydraulic resistance as a 1 m tall individual with 10 µm terminal twig conduits, and likewise, a 20 m tall individual with 21.8 µm terminal twig conduits would match them both. Because conductance scales with the fourth power of diameter, differences in terminal diameter strongly influence resistance along the entire conductive path. As a result, modest shifts in conduit diameter at the tip in principle could in principle contribute to the maintenance of conductance per unit leaf area constant with height growth.

Consistent with this possibility, some studies have documented that across angiosperm species, taller individuals do have wider twig tip conduits (Zach et al. 2010; Olson et al. 2014, 2018b; Olson et al. 2021). Detailed studies within species also show increases in terminal twig conduit diameters or conductance as individuals grow taller (Prendin et al. 2018; Echeverría et al. 2019). Remarkably, the scaling exponent for tip conduit diameter with height implied by the simple resistance‐balancing model in the previous paragraph (0.26) is very close to the empirical slope of about 0.23 reported across angiosperms (Olson et al. 2014, 2018b, 2020). This coincidence suggests that the widening of terminal conduits with height growth may not only be sufficient in principle to offset increased pathlength resistance, but may also reflect a widespread pattern in plant hydraulic design. Understanding how plants could achieve wider terminal twig conduits with height growth therefore is potentially informative regarding the ways that selection shapes plant conductive systems with height growth.

**Figure 2:**
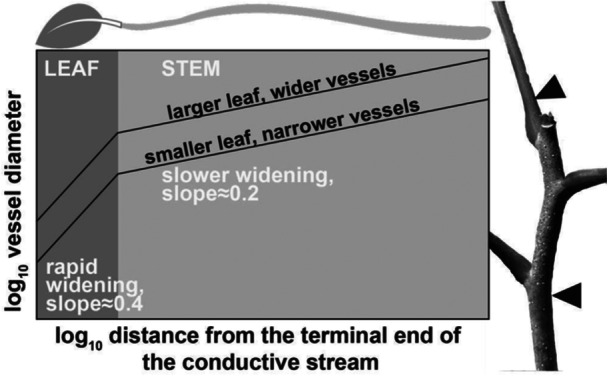
Conduit diameter tip‐to‐base leaves‐to‐stems in angiosperms. The x‐axis represents distance from the terminal end of the conductive stream (leaf tip). Vessel diameters widen rapidly through the leaf (slope ≈ 0.4, dark gray zone), then more gradually through the stem (slope ≈ 0.2, light gray zone). Petiole base vessel diameter closely predicts twig tip diameter, and because of the steep widening in the leaf, small changes in leaf length strongly affect downstream conduit diameters. This pattern implies reciprocal selective pressures between leaf size and stem conduit anatomy. Arrows on the twig photo show the sampling points: petiole base (above basal swelling) and 5 cm from the twig tip (*Bursera simaruba*).

Leaves might play a role in widening of terminal twig conduit diameters with height growth. From their very narrow termini, vessels in leaves widen toward the petiole base, coalescing into fewer, wider vessels (McCulloh et al. 2004; Gleason et al. 2018; Lechthaler et al. 2019; Rosell and Olson 2019). Whereas in stems conduit diameter *D* scales with distance from the twig tip *L* as on average *D∝L* ^0.2^, in the leaves of angiosperms, the rate of vessel diameter widening *b* is much higher. In angiosperms, vessel diameter at the petiole base (*D*_*pet*_) scales with leaf length as about *D*_*pet*_ *∝L* ^0.4^ (Coomes et al. 2008; Sack et al. 2012; Lechthaler et al. 2019; Levionnois et al. 2020; Baird et al. 2021; Levionnois et al. 2021). This means that small variations in leaf length within and across angiosperms are associated with marked differences in vessel diameter at the petiole base. It is reasonable to expect that vessel diameter at the petiole base should be closely correlated with vessel diameter at the twig tip (Figure 2).

Along these lines, Cao et al. (2022 page 8) postulated that “the vessel in the petiole is a direct smooth extension of the vessel in the twig from which it grows out.” Consistent with these expectations, the scant data available in angiosperms, from one *Acer* species, one *Fagus*, and two species of *Ficus* (Lechthaler et al. 2020; Olson et al. 2021) show that the wider petiole base vessel diameters associated with longer leaves are indeed associated with wider terminal twig conduit diameters (Figure 2). Therefore, there are reasons to suspect that leaf length positively predicts terminal twig vessel diameter.

While the tallest angiosperms do not necessarily have the largest leaves (Jensen and Zwieniecki 2013), empirical studies have found that leaf length often scales with plant height across woody angiosperm species (e.g. Jensen and Zwieniecki 2013; Price et al. 2014; Gleason et al. 2018). In turn, species with longer leaves tend to have thicker twigs and lower wood densities, consistent with the spectrum of trait covariation captured by Corner's Rules (Westoby and Wright 2003; Olson et al. 2018a). These associations suggest a suite of coordinated traits involving not just leaf size and twig diameters but potentially involving vessel diameters in the the leaves and twigs as well (Westoby and Wright 2003; Olson et al. 2018a; Fajardo et al. 2020; Larios Mendieta et al. 2021). These relationships form a set of testable predictions about how height, leaf length, wood density, and conduit diameter interact in shaping hydraulic architecture.

To explore how variation in leaf length might be involved in variation in stem hydraulic architecture, we asked a series of questions. First, does vessel diameter at the petiole base scale with leaf length across species, as previously reported? If so, can this anatomical relationship be extended further; does petiole vessel diameter reliably predict vessel diameter in the terminal twig? We then asked whether other variables known to influence xylem anatomy might help explain variation not captured by leaf traits alone. For example, does wood density, which in previous work has been found to affect the intercept of the vessel diameter versus distance‐from‐tip relationship in samples from the stem base (Olson et al. 2020), also help account for differences in twig conduit diameter? And given that taller plants tend to have longer leaves and wider conduits per unit leaf area (Echeverría et al. 2019; Anfodillo and Olson 2024), is there a general relationship between plant height, leaf length, and conduit size? We also asked whether the petiole‐twig vessel diameter relationship differed between species with simple versus compound leaves or between sites. These questions aim to identify the joint effects of leaf morphology, whole‐plant architecture, and wood properties on twig vessel diameter. In doing so, our goal is to explore the possible contribution of leaf length to ultra‐widening permeability and the maintenance of conductance to leaves with height growth.

## 2 Materials and Methods

### 2.1 Sampling

We collected samples from November 2022 to May 2023 in diverse ecological settings encompassing different regions, habitats, and growth forms. Our primary goal was to ensure the representation of a very wide span of leaf lengths because this would maximize the likelihood of detecting a relationship, if any, between leaf length and petiole base and twig vessel diameter. Field expeditions were conducted across different localities in Mexico to capture phylogenetic and habitat diversity, including deciduous tropical forest, lowland rainforest, highland desert, temperate forest, and other habitats. Cultivated samples of five species were obtained from the International *Moringa* Germplasm Collection on the coast of Jalisco, Mexico. A total of 2900 terminal twigs from 88 species representing 62 families and 32 orders of vessel‐bearing angiosperms were included (see data set in Supporting Information). We selected self‐supporting species to maximize comparability across samples. We collected branches from the upper reaches of the crown, using clippers and pole pruners for shorter plants and a Matrice 300 drone (DJI, Shenzhen, China) equipped with a DeLeaves canopy sampling tool (Outreach Robotics, Sherbrooke, Canada) for the trees 10–53 m tall.

We measured leaf length with a caliper or a tape measure. We use leaf length rather than area here because length provides a straightforward reflection of the minimum length of the hydraulic path between the terminal xylem capillaries and the petiole base. This choice allows for comparability across species with very different leaf morphologies, including both broad‐leaved and needle‐leaved species. Across most broad‐leaved species, leaf area scales approximately with leaf length squared, so in practice, both length and area tend to reflect the same underlying trend. But for the purposes of understanding hydraulic path length and the selective pressures shaping conduit diameters, leaf length would seem to be the more directly causal and universally applicable variable.

We measured plant height with a measuring tape when possible or with a TruePulse 200B laser rangefinder (Laser Technology). We assigned genera, species, and authorities based on World Flora Online (https://worldfloraonline.org/) and the International Plant Names Index (https://www.ipni.org/). We followed Angiosperm Phylogeny Group IV to assign species to orders (Chase et al. 2016).

### 2.2 Sample Processing

For consistency and comparability across samples, we standardized our measurements of vessel diameter at 5 cm from the stem apex. We chose this point because 5 cm represents a position sufficiently close to the apical meristem to capture the developmental zone where stem vasculature is plausibly very closely linked to leaf function, providing an appropriate anatomical and physiological window into the integration of leaf and stem traits, while still including well‐developed secondary xylem. We collected wood samples at a distance of 5 cm from the twig tip in all species except *Carica* (the papaya tree), for which we extended this distance to 10 cm to obtain well‐developed secondary xylem tissue suitable for anatomical analysis (see Supporting Information 1 for further details). This adjustment was necessary due to the unique developmental characteristics of *Carica*, which has very large stems and parenchymatized xylem, lacking fibers and in which the only lignified cells are vessel elements. By aligning our sampling approach with these conventions, we aim to maximize the likelihood that our data can be directly integrated into ongoing efforts to model and interpret twig‐level hydraulic behavior.

Immediately following collection, we placed all wood samples in 70% ethanol to fix tissues and preserve structural integrity for later analysis. In parallel, we removed a portion of the same wood segment to assess wood density using the water displacement method, following the protocol outlined by Williamson and Wiemann (2010; though see Fajardo 2025). To characterize leaf morphology, we measured the length of 10 to 25 fully expanded leaves in the field for each individual plant. Additionally, we excised and preserved in 70% aqueous ethanol the petiole of the longest leaf present on each sampled twig, which we later sectioned to obtain vessel diameter measurements for petiole xylem, completing the data set linking distal hydraulic structure to foliar dimensions. We used the longest leaf because it should reflect the maximum per‐leaf demand on the stem's conductive system; since the stem must at least meet this demand, twig vessel diameters are likely to reflect it.

We cut transverse sections for light microscopy from twigs at 5 cm from the twig tip and at the base of the petiole, above any pulvinus or basal swelling. For wide twigs or petioles, we cut sections with a sliding microtome, and for slender stems or petioles, we processed them for paraffin embedding. When necessary, we softened the samples in 10% ethylenediamine for 3–5 days (Carlquist 1982) before dehydrating the samples in an ethanol‐tertiary butyl alcohol series for paraffin embedding. Using a rotary microtome (Leica RM2125, Nussloch, Germany), we cut transverse sections 12–18 µm thick and stained the sections with safranin and astra blue. Following staining and dehydration in an ethanol‐xylene series, we mounted the sections in permanent mounting medium for long‐term preservation. On each slide, we randomly selected twenty‐five vessels for diameter measurement under a light microscope (Zeiss, Oberkochen, Germany). We measured vessel lumen diameter as the chord midway between the major and minor axes to account for elliptical vessels.

### 2.3 Statistical Analyses

All analyses were performed in R V. 4.4.3 (R Core Team 2020). To meet assumptions of normality and homoscedasticity and to reflect the disproportionate functional impact of changes in conduit diameter and leaf length across size ranges, all continuous variables were log10‐transformed (Kerkhoff and Enquist 2009). For example, a 10 µm increase in conduit diameter increases conductance 16‐fold when going from 10 to 20 µm, but has far less impact going from 140 to 150 µm.

We fit several linear regression models to test the relationships among mean species leaf length, petiole and twig vessel diameters, wood density, and plant height. Specifically, we tested whether petiole base vessel diameter scales with leaf length, as previously reported, and whether petiole vessel diameter predicts twig tip vessel diameter. We also evaluated whether leaf length, wood density, or plant height improved predictions of twig vessel diameter, and whether these variables interact. To test for a possible effect of leaf type or site, we fit a linear model predicting vessel diameter at the twig tip using petiole base vessel diameter, plus leaf type (simple vs compound) or site (excluding cultivated plants), and their interactions. Because conduit diameter is expected to increase causally with path length (rather than the reverse), and because leaf morphology and wood traits are treated as predictors of vessel diameter, we used OLS regression rather than Standard Major Axis (SMA) regression. In this causal framework, OLS is the appropriate method (Smith 2009; Kilmer and Rodríguez 2016).

### 2.4 Phylogenetically Informed Analyses

Finally, we explored the possibility that phylogenetically closely related species tend to resemble one another more closely than more distantly related ones. To do so, we reconstructed the phylogenetic relationships among the sampled species using a “supertree” approach implemented with the U.PhyloMaker package (Jin and Qian 2023) in R. This package allowed us to graft species in our species list to a backbone plant tree (GBOTB.extended.WP.tre), using a file with the information of genus and families to assign our species to the tree. We prepared the input files in comma‐separated values (csv) format with information on genus and families following Angiosperm Phylogeny Group IV (Chase et al. 2016). We pruned the resulting tree to include only the species in our data file. Most phylogenetic comparative methods implemented in R require an ultrametric binary tree, so we used the *fix.poly* function of the RRphylo package V 2.8.1 (Castiglione et al. 2018) to resolve the polytomies randomly. In this manner, we were able to produce an ultrametric fully resolved tree with 88 tips for the species in our database (the data are included as “Data set with species studied, leaf length, vessel diameters, wood density, and plant height” in the Supporting information)

Using this tree, we first verified whether each of the studied traits showed phylogenetic signal (i.e. the tendency for closely related species to resemble one another in their residuals more than to distantly related ones) with the phylo4d function of the phylosignal package V 1.3.1 (Keck et al. 2016). None of the characters in our database presented a statistically significant phylogenetic signal (Supporting information 1). Then, we implemented a phylogenetic generalized least squares analysis with the gls function built in the ape package (V.5.8) (Paradis et al. 2004) in R. We tested correlation structures derived from a non‐phylogenetic or “white‐noise” process (corPagel, λ = 0), Brownian motion (corBrownian), and Ornstein–Uhlenbeck (corMartins) models. We used the Akaike Information Criterion (AIC) to compare model fit and select the best model.

## 3 Results

In this study, we examined the relationships between leaf length, vessel diameter, wood density, and plant height across a diverse range of angiosperm species. Our sampling strategy was focused on finding a wide range of leaf length in localities with different climatic regimes. This allowed us to encompass a broad spectrum of plant heights, leaf lengths, and xylem conduit diameters while including representatives of major plant lineages totaling 62 families, and 31 orders of monocot and “dicot” plants (Table 1; see phylogeny in Supporting information 1. Data are provided in the file “Data set with species studied, leaf length, vessel diameters, wood density, and plant height” in the Supporting Information).

**Table 1:** Mean, minimum, and maximum values for the variables measured.

Variable abbreviation	Min	Max	Mean ± SD
Leaf length (cm) *L*	0.30 *Ixchelia mexicana*	700 *Attalea butyracea*	22.33 ± 70.88
Mean twig vessel diameter (µm) *D*_*twig*_	9.2 *Alluaudia procera*	85.8 *Xanthosoma robustum*	39.56 ± 23.60
Mean petiole vessel diameter (µm) *D*_*pet*_	3.39 *Salvia thymoides*	81.4 *Xanthosoma robustum*	28.83 ± 19.90
Wood density *WD* (g/cm^3^)	0.04 *Hedyosmum mexicanum*	6.42 *Piranhea mexicana*	0.38 ± 0.62
Plant height (m) *H*	0.25 *Peperomia obtusifolia*	52.50 *Ceiba pentandra*	8.813 ± 9.68

For detecting biological relationships, it is important to cover very wide trait ranges in the independent variables, especially at the small end of the range of variation given the disproportionate functional impact of variation in size toward the small end of the range of trait variation (Kerkhoff and Enquist 2009; Smith 2009; Olson et al. 2021). For example, one of our studied species, *Ixchelia mexicana* (Ging.) H.E. Ballard & Wahlert had a narrower vessel diameter than the mean minimum vessel diameters found in previous global studies (e.g. Echeverría et al. 2022). We also include very wide vessels, as in the large‐leaved monocot *Xanthosoma*, at nearly 86 µm in diameter at 5 cm from the twig tip. Thus, our sampling spans an exceptionally wide range of variation in the variables of interest, all tightly standardized by distance from the twig tip.

**Figure 3:**
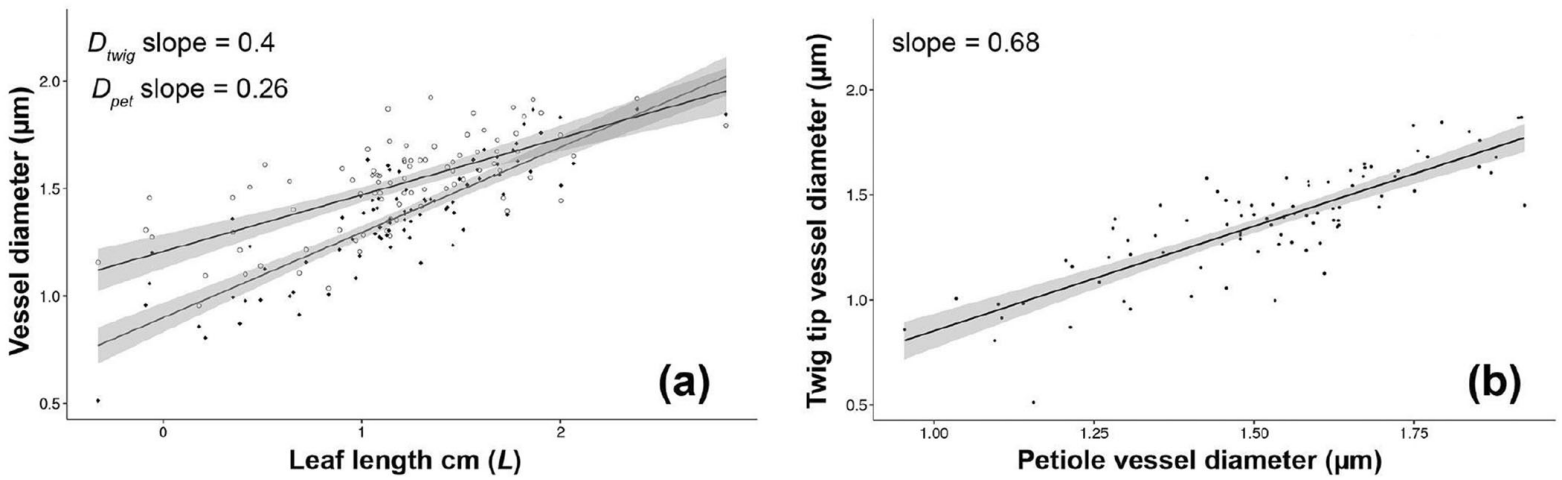
Leaf length and its effect on twig vessel diameter. (a) Vessel diameter at the base of the petiole (*D*_*pet*_) is predicted strongly by leaf length (*L*). The slope of the regression model is almost 0.4, about double the tip‐to‐base widening rate found in angiosperm stems (~0.2). (b) Vessel diameter at the twig tip is strongly predicted by vessel diameter at the petiole base.

Our results, summarized in Tables 2 and 3, are consistent with the expected relationships. We hypothesized that leaf length would strongly predict terminal twig vessel diameter, with additional influences from wood density and plant height. Congruent with previous research (Sack et al. 2012; Gleason et al. 2018; Cao et al. 2022), we found that the diameter of the vessels at the base of the petiole (*D*_*pet*_) scales with leaf length (*L*). This relationship exhibited a relatively high slope (*b* = 0.4), indicating a marked increase in vessel diameter with increasing leaf length (Table 2; Figure 3a). *D*_*twig*_ scales with *L* with a slope of 0.26 (Table 2; Figure 3a), indicating a positive but lower slope compared to *D*_*pet*_. This difference in the exponents is statistically significant (*t* = 3.298, *df *= 166.24, *p*‐value = 0.001).

**Table 2:** Scaling of vessel diameter at the petiole base *D*_*pet*_ and vessel diameter 5 cm from the twig apex *D*_*twig*_ with leaf length *L*, wood density *WD*, and plant height *H*.

Response variable	Predictor variable	*N*	*R* ^2^_adj_	*df*	*F*	Slope	Intercept	Figure
*D*_*pet*_								
	*~L*	88	0.72	86	228.4***	0.396 (0.344, 0.448)	0.898 (0.830, 0.966)	3a
	*~WD*	88	0.22	86	26.7***	−0.421 (−0.583, −0.259)	1.112 (1.003, 1.221)	—
	*~H*	88	0.16	86	17.9***	0.191 (0.101, 0.281)	1.230 (1.149, 1.312)	—
*D*_*twig*_								
	*~L*	88	0.45	86	72.9***	0.263 (0.201, 0.324)	1.210 (1.130, 1.290)	3a
	*~WD*	88	0.09	86	10.2***	−0.235 (−0.382, −0.089)	1.379 (1.280, 1.478)	4a
	*~H*	88	0.09	86	10.3***	0.126 (0.048, 0.204)	1.431 (1.361, 1.502)	5b
	*D*_*pet*_	88	0.65	86	169.4***	0.679 (0.576, 0.783)	0.592 (0.447, 0.736)	3b
*WD*								
	~*L*	88	0.24	86	29.3***	−0.271 (−0.370, −0.171)	−0.284 (−0.414, −0.154)	4b
*L*								
	~H	88	0.22	86	26.5***	0.482 (0.296, 0.668)	0.839 (0.670, 1.007)	5a

In addition to these findings, we also confirmed that vessel diameter in the twig (*D*_*twig*_) is significantly wider than that at the base of the petiole (*D*_*pet*_), indicating a continual increase in conduit diameter from the petiole into the twig (Table 2). As hypothesized, vessel diameter at the base of the petiole (*D*_*twig*_) was found to scale positively with vessel diameter in the twig (*D*_*pet*_), with a slope of 0.68 (Figure 3b). This indicates that as the diameter of petiole base vessels increases, the diameter of twig tip vessels increases as well.

**Figure 4:**
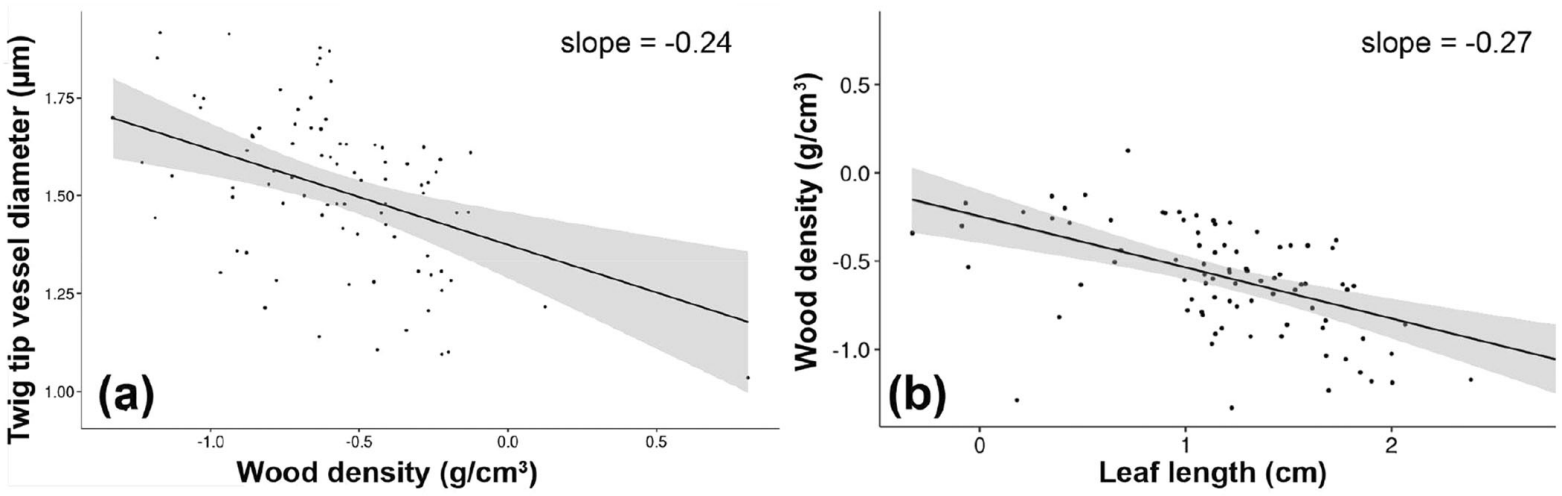
Wood density, twig vessel diameter, and leaf length. (a) Species with lower wood density have, for the same distance from the twig tip, wider vessels. (b) This pattern is expected if species with longer leaves have lower wood density, and this is indeed the case.

**Figure 5:**
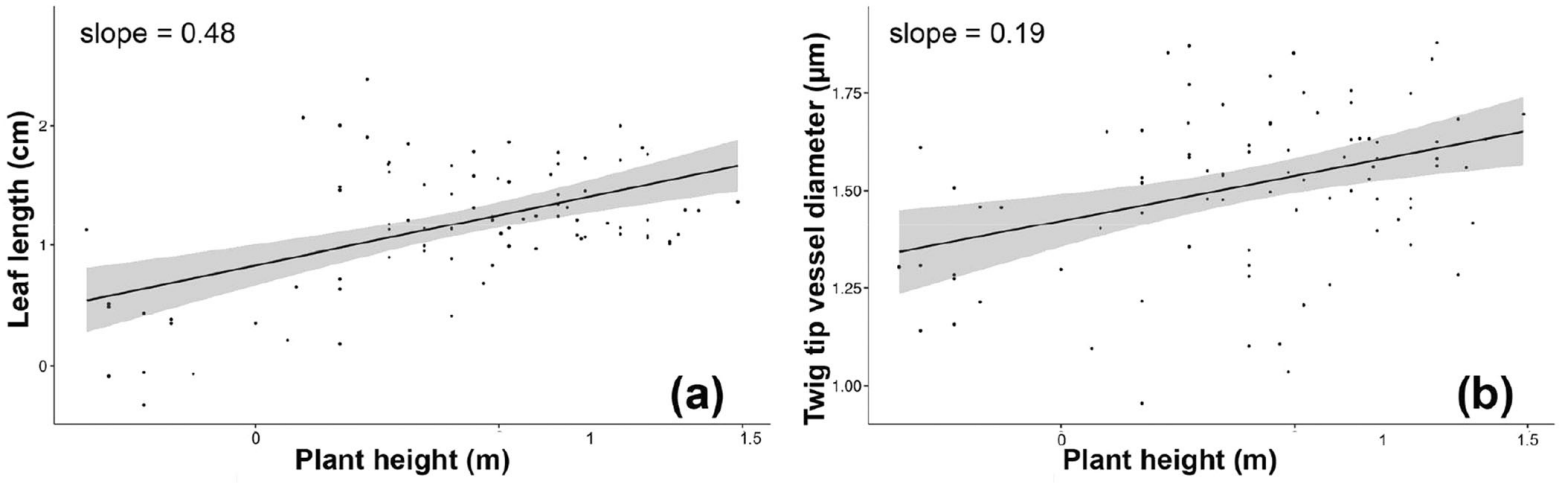
Plant height, leaf length, and twig vessel diameter. (a) Longer leaves tend to be found on taller plants. (b) Taller plants also have wider terminal twig vessels.

Other significant univariate associations were observed. Specifically, species with lower wood density tended to have wider twig vessels, as indicated by a negative regression coefficient (slope = −0.24, Table 2; Figure 4a), as well as wider petiole vessels (*D*_*pet*_) (−0.42, Table 2). We recovered the well‐documented negative relationship between wood density and leaf length (slope = −0.27, Table 2; Figure 4b) (Swenson and Enquist 2008). Additionally, plant height positively predicted leaf length (slope = 0.48; Figure 5a), suggesting that taller self‐supporting plants tend to have longer leaves. In addition to longer leaves, taller species also tended to have slightly wider vessels in both twigs (slope = 0.13; Figure 5b) and petioles (slope = 0.19, 95% CI: 0.101, 0.281; Table 2). Leaf type and site had no effect on the relationship between petiole and twig vessel diameter. Neither the slopes (compound/simple · *D*_*pet*_ *p* = 0.815; site · *D*_*pet*_ *p* = 0.1249) nor the intercepts (compound/simple *p* = 0.502; site *p* = 0.583) differed significantly.

Phylogenetically informed analyses produced results entirely congruent with standard regressions (Table 3), showing that the relationships between leaf length, vessel diameter, wood density, and plant height are robust across lineages. The best‐fitting models for the relationships between characters were consistently the ones with no effect of the phylogenetic structure (White Noise, WN), or the Ornstein‐Uhlenbeck (OU) models where the mean value of the character tends to an optimal value regardless of the lineage (Table 3). As in other studies of hydraulic traits (Olson et al. 2018b), phylogenetic signal tests revealed no significant similarities in individual traits among closely related species (Supporting Information 1), challenging the expectation that “structural” traits like leaf area are more evolutionarily conserved than “functional” traits (Ávila‐Lovera et al. 2023). Our phylogenetic generalized least squares (PGLS) analyses consistently aligned with non‐phylogenetic models (Tables 2 and 3), suggesting that shared ancestry plays a limited role in shaping the observed trait relationships. This seems entirely expected given that hydraulic pathlength exerts the same potent selective pressure regardless of ancestry. As a result, size should predict conduit diameter in a given species, not which species is closely related to it.

**Table 3:** Phylogenetic generalized least squares for vessel diameter at the petiole base *D*_*pet*_ and vessel diameter 5 cm from the twig apex *D*_*twig*_ scaling with leaf length *L*, wood density *WD*, and plant height *H*.

Response variable	Predictor variable	*N*	*df*	Slope	Intercept	Model	AICc (ΔAICc)
*D*_*pet*_							
	* ~L*	87	85	0.396 (0.344, 0.448)***	7.925 (6.781, 9.261)***	WN	−82.893 (1.38)
	* ~WD*	87	85	−0.376 (−0.519, −0.233)***	14.025 (11.221, 17.530)***	WN	3.113 (0.0008)
	* ~H*	87	85	0.083 (0.044, 0.122)***	17.020 (14.106, 20.537)***	WN	13.029 (1.27)
*D*_*twig*_							
	* ~L*	87	85	0.263 (0.203, 0.323)***	16.112 (13.456, 19.292)***	WN	−58.251 (1.81)
	* ~WD*	87	85	−0.238 (−0.364, −0.112)***	23.925 (19.658, 29.120)***	OU	−18.454 (0.82)
	* ~H*	87	85	0.056 (0.023, 0.089)**	26.665 (22.704, 31.318)***	WN	−13.349 (2.11)
	* ~D*_*pet*_	87	85	0.683 (0.583, 0.782)***	3.836 (2.787, 5.278)***	OU	−104.062 (0.14)
*WD*							
	~*L*	87	85	−0.280 (−0.392, −0.168)***	1.818 (0.391, 0.773)***	OU	48.718 (1.30)
L							
	* ~H*	87		0.210 (0.129, 0.290)***	6.867 (4.660, 10.118)***	OU	136.159 (0.01)

*Note:* We tested the relationships under an Ornstein–Uhlenbeck evolutionary model (OU, *α* = 1), a brownian motion evolutionary model (BM, λ = 1), and a white noise model (WN, λ = 0) equivalent to no effect of the phylogenetic structure in relationships between variables. The results of the best model according to AICc are shown.

## 4 Discussion

### 4.1 Variation in Leaf Length and Ultra‐Widening Permeability

Our data show that increases in leaf length lead to much wider vessels at the twig tip, due to the steep scaling between leaf length and petiole base vessel diameter (Table 2; Figure 3a) and the link between petiole and twig diameters (Figure 3b). The much steeper conduit widening in the leaves of angiosperms compared to stems leads to a striking hydraulic outcome: resistance in the leaf is nearly independent of its length (Figure 1). Using a simplified model of stacked cylindrical conduits and invoking Poiseuille's law, when widening follows an exponent of 0.4, as in leaves, resistance increases only modestly with distance, from ~0.07 units at 100 mm to 0.18 at 1000 mm. By contrast, stem conduits with a 0.2 exponent show resistance rising from ~0.16 to ~1.0 unit over the same length. This means that, as compared to stems, longer leaves add minimal resistance to the whole path, despite their longer pathlengths.

This near independence of resistance from leaf length suggests that selection can shape leaf length in response to factors other than leaf‐level hydraulic costs, including stem‐level resistance. Our data show that longer leaves are associated with wider vessels at the twig tip (Table 2; Figure 3b). Wider tip vessels should shift the entire vessel widening trajectory upward, increasing vessel diameter along the stem and reducing overall resistance. As trees grow taller, increasing path length imposes higher resistance, and while tip‐to‐base widening helps buffer this effect, it is changes in the diameter of the “starting” vessels, those at the twig tip, that unlock especially large gains in conductance (Echeverría et al. 2019). Because vessel diameter scales so steeply with leaf length, even modest increases in leaf length during height growth appear to have the potential to elevate stem‐wide permeability, and help to maintain near‐constant conductance per unit leaf area.

Our results thus help address a broader question: how might the whole plant maintain hydraulic function while allocating carbon efficiently as it grows taller? Our “stretched” model of plant construction (Anfodillo and Olson 2024) postulates that selection should favor a constant relationship between leaf area, which produces photosynthates, and metabolically active sapwood volume, which is a carbon sink. Berry et al. (2024) confirmed this isometric scaling in a detailed study of one species, and various other studies are compatible with the possibility (see Table 1 in Anfodillo and Olson 2024). If such scaling is general, then for every unit of leaf area, the same total sapwood volume must be maintained regardless of plant height. But in a taller plant, this volume is distributed over a longer distance, from the base of the trunk to the twig tip. Imagine stretching a fixed volume of sapwood into a taller column: as it lengthens, its cross‐sectional area must shrink. In other words, per unit leaf area, sapwood becomes longer and narrower as a tree grows taller. This geometric consequence implies that taller plants should have less xylem cross‐sectional area per unit leaf area, and thus fewer conduits moving water.

Selection therefore plausibly favors mechanisms that allow conductance to remain constant through increasingly narrow xylem cross sections per unit leaf area. Together with tip‐to‐base widening, widening of terminal twig conduits with height growth could allow trees to supply their leaves with fewer conduits per unit leaf area as they grow taller. In the angiosperm tree *Moringa oleifera*, terminal twig vessel diameters did increase with height, with a concomitant decrease in vessel number per unit leaf area, such that conductance per unit leaf area remained constant (Echeverría et al. 2019). Similarly, a study in *Picea abies* showed that hydraulic conductivity of apical shoots increased with tree height (Prendin et al. 2018). Given that plants almost always increase individual leaf length when growing from seedlings to adults, our results suggest that increasing leaf length plausibly serves as a key mechanism supporting ultra‐widening permeability during height growth.

### 4.2 Leaf Length, Vessel Diameter, and Wood Density

The relationship between conduit diameter and wood density appears to be indirect and shaped by the broader chain of causation described by Corner's rules. Vessel diameter variation does not account for differences in wood density (Ziemińska et al. 2013; Ziemińska et al. 2015), suggesting that even though species with long leaves tend to have wider vessels in the twig, these wider conduits are unlikely to explain their lower wood density (Figure 4a). Instead, these associations seem to emerge from the ways that longer leaves influence twig construction more generally. Longer leaves are subject to selection favoring wider spacing in reducing self‐shading (Smith et al. 2017), but because lifetime carbon gain per unit crown area is broadly similar across species (Castorena et al. 2022), plants with large leaves have roughly the same carbon budget per unit leaf area to invest in twig structure. Wider spacing between long leaves with this same carbon budget necessitates a reduction in twig tissue density, hence the negative relationship between leaf length and wood density observed across species (Figure 4b) (Swenson and Enquist 2008). This linkage drives additional patterns, such as positive relationships between leaf length, pith diameter, and bark thickness, and the negative one between leaf length and twig mechanical resistance (Olson et al. 2009; Méndez‐Alonzo et al. 2012). In twigs bearing large leaves, most of the cross‐sectional area is often occupied by pith and bark, with wood forming a relatively small fraction, again emphasizing that lower wood density stems from tissue allocation, not from conduit dimensions per se.

### 4.3 The Relationship Between Leaf Length and Plant Height

Although leaf length and plant height are almost certainly decoupled developmentally– *Gunnera* species, for instance, can produce leaves over 3 m long on stems only 30 cm tall–existing datasets on woody angiosperms consistently find a positive relationship between mean leaf length and plant height, including the present study (Table 2) (Jensen and Zwieniecki 2013; Price et al. 2014; Gleason et al. 2018). This pattern suggests that, in the context of this developmental independence, selection may often favor longer leaves in taller plants, at least across woody plants. Our findings provide a potential explanation for this trend: because leaf length predicts vessel diameter at the twig tip, increases in leaf length can enhance stem‐wide conductance by shifting the vessel widening trajectory upward. This may contribute to the observed coordination between leaf length and plant height in available woody angiosperm datasets.

### 4.4 Conclusion

Despite the linkage between leaf and stem being functionally important, surprisingly little is known about the quantitative relationship between leaf conduit diameters and the diameters of the conduits in the twigs that leaves are inserted on. Our results provide affirmative answers to the anatomical and functional questions posed at the outset, and support the idea that changes in leaf morphology plausibly propagate upstream to influence stem‐wide hydraulic architecture.

## Conflicts of Interest

The authors declare no conflicts of interest.

## Supporting information

Dataset with species studied, leaf length, vessel diameters, wood density, and plant height.

Supporting information 1.

## Data Availability

Data sharing not applicable to this article as no datasets were generated or analyzed during the current study.
