# Supplementary material for: Leaf Length Predicts Twig Xylem Vessel Diameter Across Angiosperms
Source: Plant Cell Environ. 2025 Nov 24;49(3):1330–9. doi: 10.1111/pce.70287 (PMC12873519; doi:10.1111/pce.70287)
Supplement: Supplementary file 2 — Supporting information 1. [file PCE-49-1330-s002.docx]

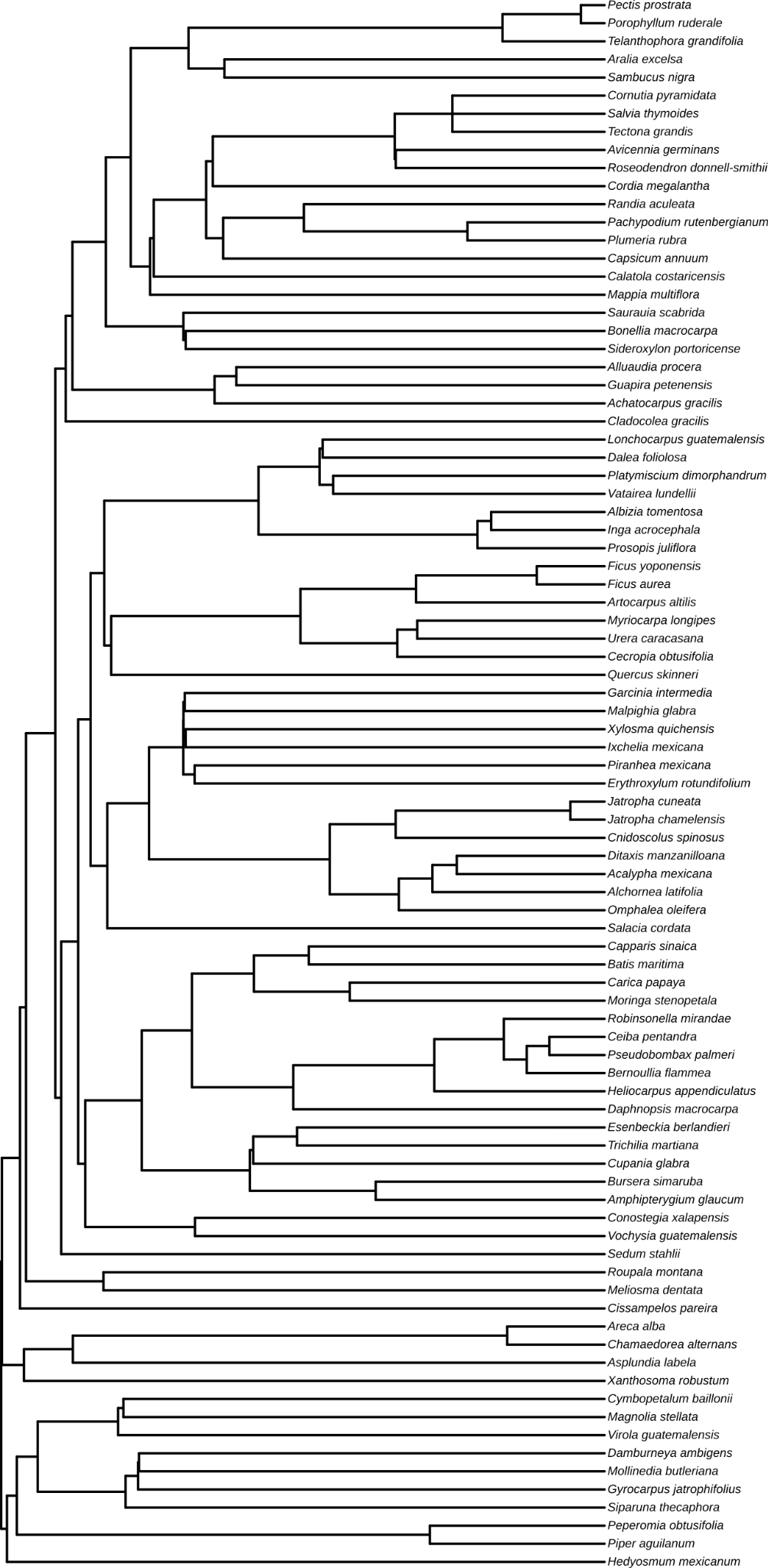


Reconstructed tree for the studied species

| **Index** | C mean  (Abouheif, 1999) Model: None | I  (Gittleman & Kot, 1990)  Model: None | K  (Blomberg et al., 2003)  Model: Brownian motion | λ  (Pagel, 1999) Model: Brownian motion |
| --- | --- | --- | --- | --- |
| **Variable** |  |  |  |  |
| *Dpet* | 0.154 p=0.02 | -0.007 p=0.238 | 0.501 p=0.251 | 0.081 p=0.80 |
| *Dtwig* | 0.22 p=0.002 | -0.007 p=0.268 | 0.459 p=0.417 | 0.363 p=0.1607 |
| *L* | 0.20 | -0.0039 | 0.455 | 0.17 |
|  | p=0.006 | p=0.136 | p=0.45 | p=0.397 |
| *WD* | 0.161 | -0.008 | 0.667 | 0.658 |
|  | p=0.01 | p=0.287 | p=0.013 | p=0.079 |

Abouheif E. (1999) A method for testing the assumption of phylogenetic independence in comparative data. Evolutionary Ecology Research 1, 895-909.

Blomberg S.P., Garland Jr T. & Ives A.R. (2003) Testing for phylogenetic signal in comparative data: behavioral traits are more labile.

Evolution 57, 717-745.

Gittleman J.L. & Kot M. (1990) Adaptation: Statistics and a null model for estimating phylogenetic effects. Systematic Biology 39, 227-241.

Pagel M. (1999) Inferring the historical patterns of biological evolution. Nature 401, 877-884.
